# In the face of climate change, will trees be…shorter?

**DOI:** 10.1093/plphys/kiad610

**Published:** 2023-11-14

**Authors:** Hannah M McMillan

**Affiliations:** Assistant Features Editor, Plant Physiology, American Society of Plant Biologists; Department of Biology, Duke University, Durham, NC 27708, USA

Current climate change models predict temperature increases anywhere from 2 °C to 5 °C over the next 75 years (Pörtner et al. 2022), which could have serious impacts on plant performance. Indeed, elevated temperatures alone can lead to decreased yield (Hatfield and Prueger 2015), increased disease susceptibility (Huot et al. 2017), and, in the case of trees, early bud burst and leaf out (Polgar and Primack 2011). Some of these effects could be beneficial; however, it is important to note that changes in one abiotic factor often affect additional abiotic factors, causing plants to experience multiple stressors simultaneously and making it difficult to predict overall plant responses to environmental changes (Fu et al. 2020; Didion-Gency et al. 2022; Petrik et al. 2022; Vitasse et al. 2022). For example, elevated temperatures often coincide with decreased soil moisture content (Breshears et al. 2005). While elevated temperature contributes to early leaf emergence in some species (Polgar and Primack 2011), low soil moisture content can delay leaf emergence (Bigler and Vitasse 2021). In many cases, the underlying cues leading to abiotic stress responses are unknown, and the combined effects of simultaneous stressors are unstudied.

**Figure 1.**
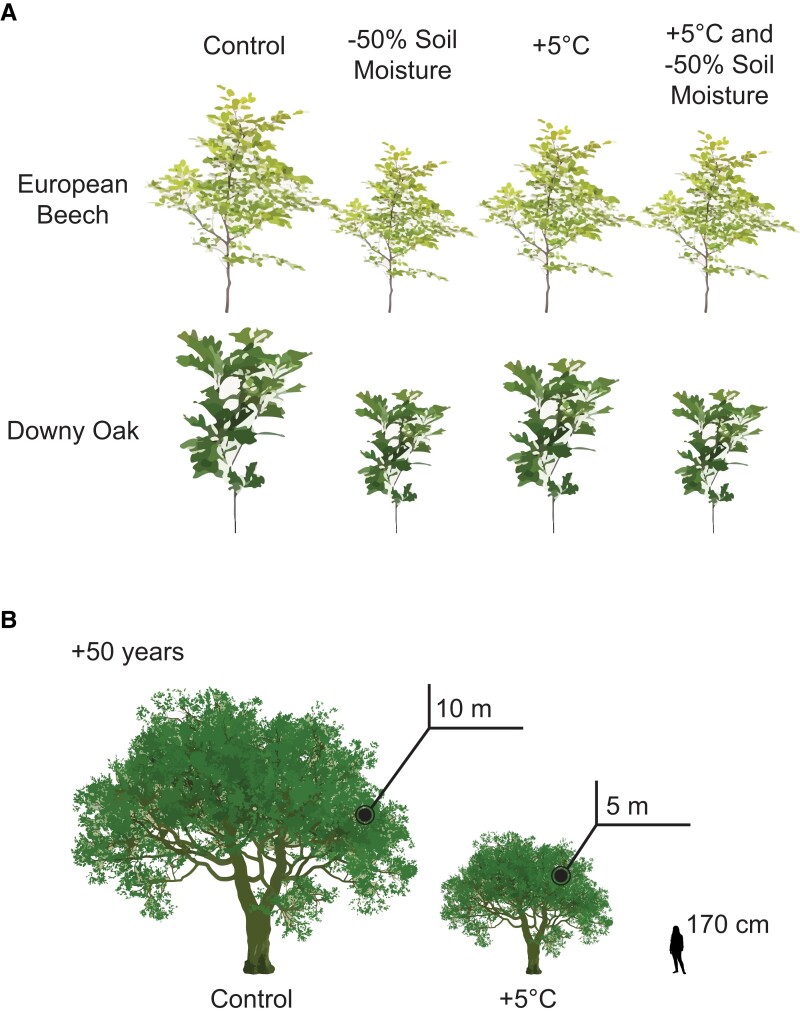
Individual and combined stress affect tree height. **A)** Visual representation of the change in overall tree height after 3 years under various stressors. Overall height is based on reported beginning tree height and change in height increment (Didion-Gency et al. 2023). **B)** Representation of oak tree height after 50 years grown under chronic warming conditions. Tree height extrapolated from reported results (Didion-Gency et al. 2023).

In this issue of *Plant Physiology*, Didion-Gency et al. reveal how the individual and combined stress of chronic warming and reduced soil moisture affect phenology, leaf-level gas exchange, and growth traits of European beech and Downy oak trees over 3 years (Didion-Gency et al. 2023). Under a chronic 5 °C increase in temperature, both oak and beech trees displayed earlier bud burst and shorter leaf development duration in spring and prolonged senescence in fall. Together, these changes in seasonal phenology timing extended the growing season of both tree species by approximately 5 days. Despite the extended season and increased opportunity to produce, store, and utilize starch, sugar, and other carbohydrates, tree height and diameter increments were significantly decreased in oak compared to control conditions (Fig. 1A). In beech trees, height and diameter increments were not affected by elevated temperature alone (Fig. 1A). These results suggest that 1) beech trees may be more resilient in the face of elevated temperatures, and 2) seemingly small changes in phenology may have large detrimental effects over the course of many years.

In contrast, a chronic 50% decrease in soil moisture content had little to no effect on tree phenology but resulted in a significant and much larger decrease in tree growth and diameter for both beech and oak by the end of the study (Fig. 1A) (Didion-Gency et al. 2023). Considering the impact of each individual stress on tree phenology and physiology, one may predict that the combination of both stressors might have an additive, detrimental effect on tree performance. Indeed, trees subjected to combined stress show decreased height and diameter increments in both beech and oak (Fig. 1A). As predicted from individual stress, oak diameter increment is decreased even further under combined stress than either individual stress though not significantly more than reduced moisture stress alone. Intriguingly, though again not statistically significant, beech height and diameter increments are slightly improved under combined stress compared to reduced moisture stress alone. These data provide an important step forward in understanding long-term impacts of combined climate change–associated stress and also highlight the complexity in predicting how global changes will affect permanent vegetation.

One particularly interesting implication of these findings is that elevated winter temperatures have dramatic impacts on spring phenology. For example, chronic winter warming raised the temperature needed for bud burst in the spring, suggesting that there were not enough chilling days to fully break dormancy (Didion-Gency et al. 2023). In this study, the elevated spring temperature compensated for the lack of dormancy, and leaf development was able to proceed. However, one could imagine that a fall or winter heat wave combined with lower-than-average spring temperatures may create a situation where trees cannot break dormancy and are unable to produce leaves or show severely delayed leaf emergence. This could have devastating long-term impacts because trees may be stunted or even die.

Although significant, the changes observed in this study in response to individual and combined stress may seem small; however, this is to be expected given the complex nature of the traits examined and the manipulation of multiple variables in the study design. Further, small changes can have dramatic impacts over the course of a tree's lifetime. Consider a tree that is 65 cm tall and grows 10 cm less under chronic warming over the course of a year, as observed in this study. After 10 years, a tree in normal conditions would grow 200 cm and be 265 cm tall. In contrast, a tree in chronically warmer conditions would grow 100 cm and be 165 cm tall—a full meter shorter than the control tree. After 50 years, whereas a control tree would be ∼10 meters tall, the tree in chronically warm conditions would be only ∼5 meters (Fig. 1B). If you expand this result to a forest of 1,000 trees, the collective height lost to warming is 5,000 meters. Considering the economic impacts this height loss would have on logging and construction industries and, perhaps more importantly, the environmental impacts on carbon capture and global climate change, relatively small height losses each year can have dramatic global effects.

Slight changes in plant growth due to abiotic stress have astounding implications over extended time scales. Although studies using single stressors are informative and can facilitate mechanistic understanding of various responses, their results may not allow us to predict phenological and physiological changes under combined stress. Results such as those presented here by Didion-Gency et al. are the first steps in understanding the long-term impacts of climate change.
